# Numerical and Experimental Analysis of Thermal, Electrical and Mechanical Aspects of Carbon-Based Composites

**DOI:** 10.3390/ma19040668

**Published:** 2026-02-10

**Authors:** Giovanni Spinelli, Vittorio Romano

**Affiliations:** 1 Faculty of Transport Sciences and Technologies, University of Benevento “Giustino Fortunato”, Via Raffaele Delcogliano 12, 82100 Benevento, Italy; 2 Open Laboratory on Experimental Micro and Nano Mechanics, Institute of Mechanics, Bulgarian Academy of Sciences, Acad. G. Bonchev Str., Block 4, 1113 Sofia, Bulgaria; 3 Department of Industrial Engineering, University of Salerno, Via Giovanni Paolo II, 132, 84084 Fisciano, Italy

Carbon-based composites have emerged as a pivotal class of multifunctional materials, distinguished by their exceptional ability to integrate mechanical reinforcement with enhanced electrical and thermal functionalities. The incorporation of fillers such as carbon fibers, carbon nanotubes (CNTs), graphene, and carbon black into polymeric, cementitious, or hybrid matrices has paved the way for advanced materials tailored for structural health monitoring, energy storage, and thermal management.

The rapid evolution of this field is evidenced by the growing number of experimental and numerical studies dedicated to the analysis of the coupled thermo-electro-mechanical response of composites, even under complex loading and environmental conditions. This progress is exemplified by the contributions collected in the MDPI Special Issue “Numerical and Experimental Analysis of Thermal, Electrical and Mechanical Aspects of Carbon-Based Composite Systems”, edited by the authors.

From a mechanical perspective, carbon-based reinforcements are renowned for significantly enhancing stiffness, strength, and fracture resistance across multiple length scales.

Research on carbon fiber-reinforced mortars and polymers has highlighted pronounced pseudo-ductile behavior, improved load redistribution, and greater damage tolerance under both monotonic and cyclic loading [1].

Specifically, the flexural and compressive responses of unidirectional and multidirectional composites highlight the critical role of fiber orientation, interfacial bonding, and matrix confinement in governing crack initiation and propagation [1,2]. These results are further supported by investigations into in-plane compression and fracture growth, where strain-rate sensitivity and progressive damage evolution significantly affect macroscopic performance [2].

Beyond structural benefits, carbon nanofillers enable electrically and thermally active networks within inherently insulating matrices. The formation of percolating conductive pathways allows for tunable electrical conductivity across several orders of magnitude, making these composites ideal for electromagnetic shielding and sensing. Investigations into epoxy-based nanocomposites filled with CNTs and graphene reveal that even minimal filler concentrations can drastically alter AC conductivity and dielectric relaxation, particularly when influenced by environmental factors such as temperature and moisture [3]. Controlling this moisture dependent electrical behavior is essential to ensuring the long-term reliability of functional insulation materials [3].

Thermal performance constitutes another vital aspect of multifunctionality. The high intrinsic thermal conductivity of graphene and CNTs, when effectively dispersed, substantially improves heat dissipation and the overall thermal stability of polymer matrices.

The inherent complexity of these systems has necessitated the use of advanced numerical modeling to complement empirical observations. Multiphysics simulations with finite elements are increasingly employed to capture the intricate interplay between mechanical deformation, electrical conduction, and thermal transport properties [4]. Such frameworks provide deep insights into local stress fields and transport phenomena that are often inaccessible through experimental means alone. Recent work by the authors underscores the necessity of integrating experimental data with numerical simulations to achieve robust predictive capabilities [5,6,7,8]. This integrated in which structural integrity and functional performance must be optimized simultaneously.

Despite these advancements, challenges remain regarding the scalability of models due to material heterogeneity and sensitivity to processing conditions—specifically filler dispersion and interfacial integrity. Furthermore, the nonlinear, history-dependent coupling between physical responses requires more sophisticated multi-physics frameworks.

Overcoming these obstacles is crucial for the deployment of reliable “smart” materials in industrial applications.

Bridging the gap between fundamental research and industrial scalability is imperative to fully realize the potential of advanced carbon-based composites.

To this end, this Special Issue has gathered contributions that advance the design, sustainable synthesis, and multiscale characterization of these systems. Moving forward, however, several key research directions must be prioritized to transition these materials from the laboratory to large-scale engineering applications.

Future research should focus on the development of standardized, eco-friendly manufacturing processes to ensure reproducibility while minimizing environmental impact through circular economy principles. Furthermore, there is a critical need for advanced data-driven approaches, such as Machine Learning and Artificial Intelligence, to accelerate the discovery of optimal filler-matrix configurations and to predict long-term durability under real-world aging conditions. Finally, investigating the dynamic, real-time coupling of multi-physical properties will be essential for the next generation of autonomous “smart” structures capable of self-sensing and adaptive thermal management.
